# Comparison of the effectiveness of botulinum toxin, dry needling, pharmacological treatment, and manual therapy for bruxism-induced myalgia: a prospective randomized study

**DOI:** 10.22514/jofph.2024.043

**Published:** 2024-12-12

**Authors:** Semiha Seda Şahin, Alanur Çiftçi Şişman, Emel Atar, Hilmi Kilaç, Elifnur Güzelce Sultanoğlu

**Affiliations:** ^1^Department of Oral and Maxillofacial Surgery, Private Practice, 34520 Istanbul, Turkiye; ^2^Department of Oral and Maxillofacial Surgery, Hamidiye School of Dentistry, University of Health Sciences, 34668 Istanbul, Turkiye; ^3^Department of Physical Therapy and Rehabilitation, Medicana Hospital, 34660 Istanbul, Turkiye; ^4^Sultan 2.Abdul Hamid Khan Educational and Research Hospital, 34660 Istanbul, Turkiye; ^5^Department of Prosthodontics, Hamidiye School of Dentistry, University of Health Sciences, 34660 Istanbul, Turkiye

**Keywords:** Botulinum toxin, Bruxism, Dry needling, Emotional stress, Exercise therapy, Myalgia

## Abstract

Bruxism is a significant phenomenon that should not be underestimated, given its prevalence and consequences. The major symptoms associated with bruxism include myalgia, decreased quality of life, and limited mandibular movements. This study aimed to evaluate and compare the effectiveness of four treatment methods for managing bruxism-related symptoms: botulinum toxin (BoNT-A), dry needling (DN), pharmacological therapy (PT), and manual therapy (MT). Eighty patients with bruxism (44 female, 36 male) were randomly assigned to four groups of 20 patients each. All therapies were administered by the same maxillofacial surgeon. Measurements were recorded at baseline (pre-treatment) and at 2, 4 and 12 weeks post-treatment. The metrics assessed included the visual analog scale (VAS) for pain, maximum painless mouth opening (MMO), and the Oral Health Impact Profile-14 (OHIP-14) questionnaire. Statistical analysis was performed using a mixed-design repeated measures two-way analysis of variance (ANOVA) to compare changes within and among the groups over time. Tukey’s multiple comparison test was applied for further analysis. The results indicated that both objective and subjective clinical outcomes were similar across all treatment groups. Considering their competitive efficiency, non-invasiveness or minimal invasiveness, and cost-effectiveness, DN, MT and PT appear to be promising alternatives for managing bruxism and its symptoms, especially in the early stages. ClinicalTrials ID: NCT06583551.

## 1. Introduction

Bruxism refers to a repetitive jaw muscle activity characterized by teeth clenching or grinding that can occur at night (sleep bruxism) or during the day (awake bruxism) [1]. According to an epidemiological study, the global prevalence of bruxism is approximately 22.22%, with sleep bruxism at 21% and awake bruxism at 23% [2]. A recent study reported that approximately 8%–31% of the general population experiences bruxism to some degree during their lifetime [3].

The etiology of bruxism is multifactorial and not fully understood; however, psychological factors are believed to play a primary role [2]. Higher stress and anxiety levels are often associated with bruxism [4]. Diagnosis of bruxism relies primarily on self-reports, partner reports, questionnaires and clinical findings, although tools such as electromyography (EMG) and polysomnography (PSG) can provide additional diagnostic support [5].

Although bruxism is not life-threatening, its symptoms can profoundly influence the quality of life (QoL). Oral health plays an important role in an individual’s general health. Oral health issues can result in changes in oral manifestations, thereby impacting all aspects of life, both physically and psychologically. In addition to oral health problems such as mechanical wear of the teeth, tooth hypersensitivity or fractures, damage to restorations, and dental implants, bruxism can cause myalgia, arthralgia in the temporomandibular joint, stiffness and hypertrophy of the masticatory muscles, headaches, disrupted sleep and fatigue. Oral health-related quality of life (OHRQoL) measures how oral health—along with functional, psychological and social factors, as well as pain or discomfort—influences an individual’s overall well-being, making it a valuable research topic for oral health researchers. Previous studies have demonstrated that individuals with bruxism tend to have lower OHRQoL compared to non-bruxists [6, 7]. Myalgia in the masticatory muscles is a major symptom associated with bruxism [8].

Previous studies have suggested that limited mouth opening and jaw movements, as well as a feeling of fatigue or stiffness in the muscles, are also directly related to bruxism [9].

Treatment options range from non-invasive approaches, such as cognitive behavioral therapy (CBT), biofeedback therapy (BFT), oral appliances (OAs), oral rehabilitation through correction of malocclusion, manual therapy (MT), and pharmacological treatment (PT), to interventional therapies, such as dry needling (DN), local anesthetic injections, and botulinum toxin (BoNT-A) injection [10, 11, 12]. Although each treatment functions through distinct mechanisms, the primary goal of all of them is to reduce muscle hyperactivity and manage pain [7, 13, 14].

OAs are simple-to-make dental devices commonly used for bruxism. Although they are not curative, they function as a protective barrier against the dental, oral and muscular consequences of bruxism [15]. A recent systematic review classified the certainty of their effectiveness as low to moderate [16]. Their advantages include being non-invasive and not reducing bite forces. One disadvantage of these appliances is aging due to exposure to oral fluids, temperature changes and constant contact with the teeth during grinding. This can lead to wear and surface changes, potentially causing OAs to become dysfunctional [17]. Another side-effect is occlusal alterations—including open bite, particularly with long-term use, defined as more than 3 years [11]. Moreover, the use of OAs may be limited in some patients due to factors such as gag reflex [18].

CBT and BFT have also attracted research attention due to their non-invasive nature. CBT uses psychological techniques to shift negative thought patterns and behaviors to positive ones. However, it has been reported as ineffective in reducing muscle activity [16]. While no adverse events have been reported in studies of CBT and BFT, there is limited evidence supporting their effectiveness [11].

BoNT-A injection is commonly used to treat conditions associated with muscular hyperactivity by inhibiting the release of acetylcholine, a neurotransmitter, into the presynaptic space, thus reducing muscle contractions [19]. It is a recommended treatment option for bruxers, especially those seeking earlier symptom relief [20].

DN is a therapeutic technique that involves inserting a fine monofilament needle into the myofascial trigger points (MTrPs)—irritable nodules within the taut bands of hypertonic muscle fibers. The goal of this technique is to relieve muscle tension and restore painless muscle function without the use of any additional substances [21, 22].

PT for bruxism involves the use of sedatives, anxiolytics, tranquilizers, anti-inflammatory drugs, antidepressants, proton pump inhibitors, anti-convulsants, anti-hypertensives and muscle relaxants, which act on specific pathways through their active ingredients [9, 11, 23].

MT is a physical therapy approach that uses hands-on techniques to relieve pain, increase jaw range of motion (ROM), reduce soft tissue inflammation or restrictions, and induce muscle relaxation. The goal is to maximize functional movement without limitation or pain and to alleviate overall discomfort [5, 24, 25].

There is currently no specific treatment for bruxism, but its symptoms can be managed [26]. While numerous studies have explored the use of BoNT-A in bruxism management, there is limited consensus on alternative treatment methods [21]. Additionally, previous studies have focused on comparisons with sham treatments or only two interventions at a time, lacking a comprehensive approach [13, 14, 21, 27, 28, 29].

This randomized clinical study aims to evaluate and compare the effectiveness of BoNT-A, DN, PT and MT by assessing pain (myalgia), maximum pain-free mouth opening (MMO) and OHRQoL, with the goal of enhancing professional awareness of treatment options for bruxism. To the best of our knowledge, no previous study has compared the effectiveness of these treatment methods in patients with bruxism. Our null hypothesis states that all treatment types would be equally effective in terms of early outcomes.

## 2. Materials and methods

The study was planned as a prospective randomized clinical trial adhering to CONSORT 2010 guidelines. The study was conducted following the Helsinki Declaration principles. This study was approved by the University of Health Sciences Hamidiye Clinical Research Ethics Committee (document number 20/120) and is registered at ClinicalTrials.gov under ID NCT06583551.

### 2.1 Study design

The following subjects were eligible for study inclusion: adult patients aged 18–65 years with complete dentition, classified as American Society of Anesthesiologists (ASA) physical status ASA I or ASA II, experiencing moderate to severe pain in the masticatory muscles related to bruxism, not previously treated for bruxism, and diagnosed with bruxism. The exclusion criteria were as follows: the presence of temporomandibular joint disorder, having dentures, known allergy to botulinum toxin, pregnancy, neuromuscular disease, and chronic use of muscle relaxant medication within the last three months. Bruxism was diagnosed using a combination of questionnaires and clinical findings. Pintado criteria were applied for assessing awake bruxism (Table 1), and a clinical findings checklist defined by the American Academy of Sleep Medicine was applied for sleep bruxism [30, 31] (Table 2).

**Table 1.** Pintado questionnaire for the diagnosis of awake bruxism [30].

Questions *Positive bruxism if at least 2 positive answers.	Yes	No
Has anyone ever heard you grinding your teeth during the night?		
Do you feel your jaw tired or sore when you wake up in the morning?		
Do you feel your teeth or gum always sore when you wake up in the morning?		
Have you ever had headache temple pain when waking up in the morning?		
Are you aware of grinding your teeth during daytime?		
Are you aware of clenching your teeth during daytime?		

**Table 2.** Clinical findings defined by the American Academy of Sleep Medicine used to diagnose sleep bruxism [31].

Declaration of teeth grinding or clenching (self reported or reported by a relative) + the presence of at least one of the clinical symptoms:	Yes	No
Abnormal wear on teeth or restorations due to teeth grinding		
Temporary jaw and muscle pain or fatigue in the orofacial area		
Temporary jaw locking, especially in the mornings		
Temporal headache		

The sample size estimation was based on the average pain scores of previous studies [32, 33] and performed using the G*Power 3.1.9.2 software (University of Düsseldorf, Düsseldorf, NRW, Germany). The following parameters were considered: (a) test power of 0.9, (b) significance level of 0.05, and (c) effect size of 0.4. The calculus was conducted using an *F* test ANOVA or four groups, with three iterations. Based on these standards, 16 participants per group would be sufficient to detect statistically significant differences. However, considering possible dropouts, 20% was added to each group. Thus, the final sample size comprised 80 individuals (44 female, 36 male), which were randomly divided into four groups of 20 patients each according to the treatment type: BoNT-A, DN, PT and MT.

The study was designed as a four-armed parallel, open-label blinded, before–after (pre–post) study. Slot randomization was employed for group allocation to enhance the reliability and validity of the study results. To improve blinding, the patients in each group were informed only about the treatment they were set to receive. An informed consent form was obtained from all participants. All treatments were performed by the same maxillofacial surgeon, and a second blinded operator conducted all measurements. A third blinded operator was responsible for evaluating the data. Table 3 presents a detailed description of the treatments (Figs. 1,2,3).

**Table 3.** The detailed description of the treatment protocols applied.

Treatment	Equipments	Application
BoNT-A Group 1 (n = 20)	Botox lyophilized powder, Type A	A single session of BoNT-A injection was administered intramuscularly under anatomo-topographic guidance, with the injection sites premarked for safety, according to a previous study [34] (Fig. 1). A total of 50 IU was injected bilaterally, within the masseter muscles (30 IU), and within the anterior temporalis muscles (20 IU).
DN Group 2 (n = 20)	Plastic cylindrical guided sterile dry needles with a length of 25 mm and a diameter of 0.25 mm	Myofascial trigger points (MTrPs)—hypertonic and irritable nodules within taut bands of the muscle—were identified by palpation. Needles were bilaterally inserted to a depth of 5 mm, rotated twice clockwise, and then removed after remaining in the MTrPs for a total of 20 minutes. Three sessions were conducted at one-week intervals (Fig. 2).
PT Group 3 (n = 20)	A combination of metacarbamol (380 mg) and paracetamol (300 mg)	The prescribed dosage was 2 tablets, taken 3 times a day, for a duration of 3 weeks.
MT Group 4 (n = 20)	Glycerin cream to lubricate the skin.	Facial massage and stretching maneuvers for the masseter and temporalis muscles were performed bilaterally for 20 minutes a day over a duration of 3 weeks (Fig. 3).

*BoNT-A: botulinum toxin; DN: dry needling; PT: pharmacological therapy; MT: manual therapy.*

**Fig. 1.** BoNT-A injection points in masseter and temporalis muscle.

**Fig. 2.** Application of DN.

**Fig. 3.** Application of MT.

The following parameters were assessed at baseline (prior to treatment) and at three follow-up appointments scheduled at 2, 4 and 12 weeks after the initial treatment:

- Pain at rest and at chewing: Average pain levels from the initial treatment to each follow-up session were measured using a Visual Analog Scale (VAS) ranging from 0 to 10, where 0 indicates no pain and 10 represents the worst pain the patient has ever experienced.

- MMO: This was measured with the TheraBite ruler (TheraBite Range of Motion Scale, Atos Medical, England) (Fig. 4).

**Fig. 4.** Measurement of maximum painless mouth opening (MMO) by TheraBite ruler (TheraBite Range of Motion Scale, Atos Medical, England).

- OHRQoL: This was measured using a validated native version of Oral Health Impact Profile-14 (OHIP-14) questionnaire to evaluate the impact of oral health problems caused by bruxism on an individual’s life [35]. This questionnaire comprises 14 questions that assess the following situations: functional limitation, physical pain, psychological discomfort, physical disability, psychological disability, social disability and handicap. For each question, the patient must choose one of the following answers—0 = never, l = hardly ever, 2 = occasionally, 3 = fairly often, or 4 = very often; the higher the total score, the lower the individual’s OHRQoL. High scores from the OHIP-14 questionnaire indicate poor OHRQoL.

### 2.2 Statistical analysis

In the statistical analysis, the results recorded at baseline (before treatment) were compared with those observed at follow-ups. Moreover, the four groups were compared to verify a possible statistically significant difference among therapies. Statistical analysis of the demographic data among the groups was assessed with one-way ANOVA and Chi-square test. All data for groups were expressed as means ± standard deviation (SD) and assessed for normal distribution with the skewness and kurtosis coefficients. Owing to sufficient sample size, one-way ANOVA was used to analyze the parametric data. A mixed-design repeated measures two-way ANOVA test was used to observe the differences within and among the groups over time. This was followed by Tukey’s multiple comparison test. All analyses were performed using SPSS for Windows (release 21.0, SPSS Inc., Chicago, IL, USA), with a 5% significance level.

## 3. Results

The distribution of demographic data is shown in Table 4. No statistically significant difference was found among the study groups in terms of age, sex and systemic status. According to the skewness (within ±3.00) and kurtosis (within ±10.00) values, the data were considered to be normally distributed. No statistically significant difference was found among the groups in terms of VAS, MMO and OHIP scores before treatment (*p* > 0.05). For all groups, the mean VAS score for pain at baseline was found to be statistically significantly higher than that at three follow-up appointments (*p* < 0.05). In the intra-group comparisons according to the follow-up appointments, multiple comparison tests indicated that the origin of the differences were 4th week in the DN and MT groups in terms of VAS. MMO and OHIP increased in all groups, while no statistically significant difference was found between baseline and follow-up appointments in any group (*p* > 0.05). In addition, no statistically significant difference was found among the groups in terms of VAS, MMO and OHIP scores (Table 5).

**Table 4.** Statistical analysis of the demographic data by One-way analysis of variance and Chi-Square test.

Variable		Group (n = 20)								
		BoNT-A		DN		PT		MT		*p*
Age		36.90 ± 13.54		32.50 ± 10.47		36.75 ± 12.45		30.20 ± 11.99		0.094
Sex										
	Male	n = 7	35%	n = 10	50%	n = 9	45%	n = 10	50%	0.750
	Female	n = 13	65%	n = 10	50%	n = 11	55%	n = 10	50%	
Systemic diseases										
	No	n = 16	80%	n = 15	75%	n = 12	60%	n = 14	70%	0.545
	Yes	n = 4	20%	n = 5	25%	n = 8	40%	n = 6	30%	

*BoNT-A: botulinum toxin; DN: dry needling; PT: pharmacological therapy; MT: manual therapy.*

**Table 5.** Comparison of the data in terms of follow-up appointment and treatment type by One-way repeated measures ANOVA and Tukey’s multiple comparison test.

Assessment	Follow-up appointment	Group				*p*
		BoNT-A	DN	PT	MT	
VAS						
	Baseline	6.60 ± 2.11	6.15 ± 2.01	5.15 ± 2.32	6.05 ± 2.01	0.807
	2 weeks	3.55 ± 2.09	4.50 ± 1.85	4.00 ± 2.03	3.55 ± 2.42	
	4 weeks	2.80 ± 1.91	3.40 ± 1.98	3.20 ± 2.07	3.55 ± 2.72	
	12 weeks	3.25 ± 2.61	4.15 ± 2.08	3.75 ± 2.63	3.50 ± 3.27	
	*p*	<0.001	<0.001	0.002	<0.001	
MMO						
	Baseline	39.55 ± 5.53	43.05 ± 7.10	37.65 ± 4.21	45.00 ± 4.86	0.530
	2 weeks	42.05 ± 7.54	43.70 ± 6.63	39.70 ± 6.13	44.85 ± 4.76	
	4 weeks	44.00 ± 6.48	44.15 ± 7.21	41.05 ± 7.66	44.60 ± 5.54	
	12 weeks	42.95 ± 6.51	44.40 ± 7.75	40.80 ± 6.76	44.25 ± 5.68	
	*p*	0.070	0.581	0.132	0.884	
OHIP-14						
	Baseline	12.05 ± 8.88	12.75 ± 7.93	13.30 ± 6.67	14.70 ± 9.65	0.816
	2 weeks	12.10 ± 7.06	13.55 ± 8.01	12.25 ± 7.32	14.95 ± 10.26	
	4 weeks	15.95 ± 12.60	12.90 ± 7.64	11.95 ± 9.02	15.10 ± 10.13	
	12 weeks	16.95 ± 9.32	13.95 ± 9.62	13.95 ± 7.65	15.30 ± 8.25	
	*p*	0.020	0.661	0.980	0.632	

*BoNT-A: botulinum toxin; DN: dry needling; PT: pharmacological therapy; MT: manual therapy; VAS: Visual Analog Scale; MMO: maximum pain-free mouth opening; OHIP-14: Oral Health Impact Profile-14.*

Seven patients in the BoNT-A group reported discomfort at the injection sites, and one patient in the DN group reported itching at the needle entry site postoperatively. No allergic or any other side-effects or adverse events occurred that warranted the exclusion of any patients from the study.

## 4. Discussion

The data obtained in this study supported null hypothesis, indicating no significant differences among BoNT, DN, PT and MT in terms of both subjective outcomes, such as pain and OHRQoL in the early period, and objective outcomes, such as MMO.

Some previously proposed randomized controlled trials (RCTs) have evaluated the effectiveness of intramuscular BoNT-A injections into the masseter and anterior temporalis muscles in relieving pain. Jadhao *et al*. [28] compared BoNT-A injections (30 IU for the masseter and 20 IU for the anterior temporalis muscle), saline injections, and a control group with no injection (n = 8). They assessed pain at baseline, 1 week, 3 months and 6 months, finding greater pain relief in the BoNT-A group [28]. Guarda-Nardini *et al*. [14] also examined BoNT-A (with the same dosage) versus saline solution (n = 10) for reducing bruxism-induced pain and myofascial pain. They assessed patients at baseline, 1 week, 1 month and 6 months, reporting greater improvements in both objective (range of mandibular movements) and subjective (pain at rest and during chewing) measures in the BoNT-A group compared to the placebo [14]. De la Torre De la Torre Canales *et al*. [36] compared the mandibular range of motion in 80 female patients (n = 20) receiving saline or BoNT-A injections at varying doses (stated as low, medium and high). All BoNT-A groups showed significant improvement at 28 and 180 days post-treatment, regardless of the dose [36]. In contrast, Ayala *et al*. [27] compared BoNT-A injections in the masseter muscle with saline in 14 female patients (n = 7) with painful temporomandibular dysfunction (TMD), finding that both treatments were equally effective in reducing perceived pain after 30 days. Our study aligns with these RCTs by using a questionnaire for bruxism diagnosis, follow-up periods, BoNT-A doses and injection sites, and pain outcomes. However, most of the studies had smaller sample sizes and compared BoNT-A with sham treatments, except for one [36].

The effectiveness of DN in the orofacial region has also been explored. Blasco-Bonora *et al*. [37] applied DN to MTrPs in the masseter and temporalis muscles in bruxists (n = 17). Evaluations were conducted before treatment, immediately after treatment, and at 1-week follow-up, and an improvement in pain, tenderness and jaw opening was reported [37]. Fernández-Carnero *et al*. [29] compared DN applied to masseter MTrPs with sham treatment in 12 female patients, reporting a significant increase in maximum jaw opening 5 minutes after the intervention. Similarly, Dib-Zakkour *et al*. [21] found that DN significantly reduced facial pain and increased MMO 10 minutes after treatment in patients (n = 18) with myogenic temporomandibular disorder. Arnoni *et al*. [38] reported decreased pain and improved mandibular mobility after 7 days of DN in the masseter muscles (n = 21). In all these studies, assessments were conducted immediately or shortly after the intervention, and DN was compared with sham treatment in all except two studies [37, 38].

To the best of our knowledge, only one study has compared DN with PT. González-Perez *et al*. [39] compared DN of the lateral pterygoid muscle with oral methocarbamol–paracetamol combination therapy (n = 18), evaluating patients before treatment and at 2 and 8 weeks post-treatment. They found DN to be more effective than methocarbamol–paracetamol therapy in reducing pain and increasing MMO [39]. In our study, the same treatment protocol was used: three sessions of DN and three weeks of medication with the same pharmacological agents. However, no statistically significant difference was found among the groups, which may be attributed to the longer follow-up period.

MT has long been a key approach in managing musculoskeletal disorders, although its effectiveness remains debated [40, 41]. Guarda-Nardini *et al*. [41] compared the short-term effectiveness of BoNT-A and MT for myofascial pain (n = 15). They assessed maximum pain levels (VAS) and ROM at baseline, end of treatment, and three-month follow-up. Both treatments showed significant improvement over time, with MT slightly more effective at reducing subjective pain and BoNT-A injections slightly better at increasing jaw ROM [41]. Other studies have explored MT in combination with CBT, such as psychological counseling [42] and sleep hygiene advice [43]. These studies, with sample sizes of 13 and 12, had follow-up periods of 6 weeks and 10 days, respectively. One study [42] reported significant improvements in MMO and pain with combined MT and CBT, while another [43] concluded that the effectiveness of MT was limited. In our study, MMO improved from baseline to follow-up across all groups, but the difference was not statistically significant. This is attributable to the fact that the MMO was already within the physiological range for all groups. In addition, intragroup comparisons revealed that pain scores at the 4th week were significantly lower in both the DN and MT groups and remained below baseline levels at the 12th-week follow-up. This suggests that three sessions of DN and three weeks of MT, administered at one-week intervals, resulted in effective pain control by the end of the treatment and contributed to sustained pain management after treatment completion. The efficacy of DN is attributable to the biochemical effects of stimulating pain receptors at the trigger points, while that of MT may be related to the resolution of local ischemia [38, 44, 45, 46].

Bruxism has been linked to higher stress levels and worse OHRQoL [47]. In this study, the OHIP-14 questionnaire was used to assess the relationships among bruxism, emotional stress and QoL. Rayegani *et al*. [46] conducted an RCT comparing DN and physiotherapy (n = 14), with evaluations at baseline and one month after treatment. Both treatments were found to reduce pain and improve QoL [46]. Similarly, our study observed improvements in OHRQoL but no significant difference was found among the groups.

The treatments applied in this study have potential adverse effects, although they are mostly temporary. BoNT-A injections into the masticatory muscles, while effective, can be associated with complications such as alterations in muscle histology, muscular and neurogenic atrophy, reduced muscle fiber diameter and mass, decreased masticatory force, and reduced bone volume in the condyloid and coronoid processes, especially at higher doses. Other potential issues include swallowing difficulties, temporary facial muscle paralysis, reduced electromyographic activity, and diminished contralateral movements of the mandible [19, 48]. DN can also have minor complications, including pain during or after the procedure, bleeding, and bruising—typical responses to needle insertion. Major complications are rare but can include nerve injury, infection, excessive symptom exacerbation, drowsiness or forgotten needles. Additionally, fainting, dizziness and nausea can occur, often due to vasovagal responses [49]. These symptoms are not unique to DN and are commonly associated with vasovagal responses in patients undergoing procedures involving needle sticks. In addition, a common issue with DN is the temporary decrease in pain tolerance immediately after the procedure, usually lasting less than 72 hours. To prevent patient dissatisfaction and ensure continued adherence to treatment, it is important to inform the patients about these potential side-effects in advance [50]. MT is also associated with certain temporary adverse effects, such as soreness in muscles, increased pain and stiffness [51]. PTs may vary in side-effects, including nausea, gastrointestinal issues, constipation, abdominal pain, diarrhea, decreased sleep quality, dry mouth, dizziness, symptomatic hypotension, decreased systolic blood pressure, and blurred vision, depending on the specific drug used. The extended use of some PTs may pose safety concerns due to potential side-effects or risks of dependency [52]. In the study, aside from discomfort at the injection sites in the BoNT-A group and itching at the needling sites in the DN group, no other side-effects were reported in any of the patients.

This study has several limitations. Although the follow-up period was consistent with previous studies, it may still be considered relatively short. Nonetheless, the necessity of collecting data on the comparison of different treatments for patients, along with the challenges in recruiting participants for specific treatments, may justify the early presentation of data to minimize the risk of dropouts during successive follow-up assessments.

Given the complex etiology of bruxism, treatment effectiveness should be tailored to the specific agent and individual patient needs. This study aimed to explore the management of bruxism within the context of its consequences. Additionally, bruxism was diagnosed based on self-reported symptoms combined with clinical findings confirmed through professional examination and patient questionnaires. EMG and PSG, considered gold standards for objective diagnosis, were not used, as these instruments may be limited in certain clinical settings. Additionally, instrumental approaches are less preferred in bruxism research due to the need for specialized equipment, limited accessibility, and their relative impracticality in clinical practice [53, 54].

A wide range of treatments is currently being investigated for managing the clinical consequences of bruxism, but there is no definitive evidence indicating the most effective treatment [52]. The study focused on interventions feasible within our clinical setting and did not include alternatives such as CBT, BFT or OAs.

As in some previous studies, there was no placebo group included in this study. In before–after study designs, a control group may not be present [55]. Frisaldi *et al*. [56] suggested that placebos have limited benefits in studies with continuous subjective outcomes, such as pain. Similarly, Hróbjartsson and Gøtzsche found limited evidence supporting the clinical significance of placebo interventions [57]. Furthermore, it is important to consider the potential risk of a nocebo effect, where a condition may worsen following placebo administration [56].

## 5. Conclusions

BoNT-A, DN, PT and MT did not exhibit significant superiority over each other in the management of bruxism during the 3-month follow-up period. Given their comparable early-stage clinical outcomes, these treatments may be considered interchangeable. It is crucial for clinicians to have detailed information on the available methods for managing bruxism and its consequences, including their respective advantages and disadvantages, to make informed, evidence-based decisions when selecting the most appropriate treatment option.
